# How much of the difference in life expectancy between Scottish cities does deprivation explain?

**DOI:** 10.1186/s12889-015-2358-1

**Published:** 2015-10-16

**Authors:** R. Seaman, R. Mitchell, R. Dundas, AH Leyland, F. Popham

**Affiliations:** Medical Research Council Social and Public Health Sciences Unit, University of Glasgow, Glasgow, UK; Centre for Research on Environment, Society and Health, Institute of Health and Wellbeing, University of Glasgow, Glasgow, UK

**Keywords:** Scotland, Life expectancy, Mortality, Deprivation, Place effects

## Abstract

**Background:**

Glasgow’s low life expectancy and high levels of deprivation are well documented. Studies comparing Glasgow to similarly deprived cities in England suggest an excess of deaths in Glasgow that cannot be accounted for by deprivation. Within Scotland comparisons are more equivocal suggesting deprivation could explain Glasgow’s excess mortality. Few studies have used life expectancy, an intuitive measure that quantifies the between-city difference in years. This study aimed to use the most up-to-date data to compare Glasgow to other Scottish cities and to (i) evaluate whether deprivation could account for lower life expectancy in Glasgow and (ii) explore whether the age distribution of mortality in Glasgow could explain its lower life expectancy.

**Methods:**

Sex specific life expectancy was calculated for 2007–2011 for the population in Glasgow and the combined population of Aberdeen, Dundee and Edinburgh. Life expectancy was calculated for deciles of income deprivation, based on the national ranking of datazones, using the Scottish Index of Multiple Deprivation. Life expectancy in Glasgow overall, and by deprivation decile, was compared to that in Aberdeen, Dundee and Edinburgh combined, and the life expectancy difference decomposed by age using Arriaga’s discrete method.

**Results:**

Life expectancy for the whole Glasgow population was lower than the population of Aberdeen, Dundee and Edinburgh combined. When life expectancy was compared by national income deprivation decile, Glasgow’s life expectancy was not systematically lower, and deprivation accounted for over 90 % of the difference. This was reduced to 70 % of the difference when carrying out sensitivity analysis using city-specific income deprivation deciles. In both analyses life expectancy was not systematically lower in Glasgow when stratified by deprivation. Decomposing the differences in life expectancy also showed that the age distribution of mortality was not systematically different in Glasgow after accounting for deprivation.

**Conclusions:**

Life expectancy is not systematically lower across the Glasgow population compared to Aberdeen, Dundee and Edinburgh combined, once deprivation is accounted for. This provides further evidence that tackling deprivation in Glasgow would probably reduce the health inequalities that exist between Scottish cities. The change in the amount of unexplained difference when carrying out sensitivity analysis demonstrates the difficulties in comparing socioeconomic deprivation between populations, even within the same country and when applying an established ecological measure. Although the majority of health inequality between Glasgow and other Scottish cities is explained by deprivation, the difference in the amount of unexplained inequality depending on the relative context of deprivation used demonstrates the challenges associated with attributing mortality inequalities to an independent ‘place effect’.

**Electronic supplementary material:**

The online version of this article (doi:10.1186/s12889-015-2358-1) contains supplementary material, which is available to authorized users.

## Background

Scotland is the country with the lowest life expectancy in Western Europe [1] with the city of Glasgow having particularly low life expectancy [2]. Research has long established the association between socioeconomic deprivation and inequalities in mortality with area based measures being utilised to capture the multiple dimensions of deprivation at the contextual level [3, 4]. Although Glasgow’s socioeconomic deprivation profile is extreme- it contains more than 40 % of the most deprived areas in Scotland and over 50 % of the population of Glasgow live in the most deprived areas [5]- it has been suggested that deprivation alone cannot account for its high rates of mortality [6, 7]. For example, deprivation does not account for Glasgow’s relatively higher mortality rates when compared to English cities with similar levels of deprivation. An excess of deaths has been found across all levels of socioeconomic deprivation in Glasgow, compared to Manchester and Liverpool, with a 15 % excess even in the city’s most affluent areas [6]. Glasgow’s mortality profile also ranks poorly relative to European regions which experience similar levels of socioeconomic deprivation and comparable economic trends following post-industrial decline [8].

The inability of Glasgow’s deprivation profile to account for its high death rate suggests that an additional, but to date unknown factor, over and above socioeconomic deprivation, affects health in Glasgow [9, 10]. The proportion of Glasgow’s relatively high mortality rate which is not accounted for by socioeconomic deprivation has been labelled the “Glasgow effect” [7]. This “Glasgow effect” has been primarily identified and discussed in comparison with cities in England which have similar deprivation profiles. Glasgow also has the lowest life expectancy of all Scottish cities. However, whether Glasgow has an unexplained excess mortality relative to Scottish cities is less clear.

Therefore it has not yet been established if the “Glasgow effect” should be understood as representing a “Scottish effect” [10] – the inability of deprivation to explain Scotland’s excess mortality over England and Wales - or rather as something distinct to the city of Glasgow [7]. If socioeconomic deprivation was unable to explain Glasgow’s excess mortality in comparison to Scottish cities, it would imply that there is indeed, something distinct about the city’s health within Scotland.

The finding that mortality varies from place to place is not unique to Scotland. Studies from across the UK [11, 12] and other countries [13] show systematic differences in mortality between geographical areas. The debate that is central to most of these studies is therefore how much of the health difference is due to individual level factors versus how much is due to contextual level factors, which reflects the extent to which health is seen to be shaped by individual behaviours and lifestyles, and by structural and environmental conditions [4, 14]. Yet fully accounting for contextual level factors, over and above socioeconomic deprivation, that may be driving higher mortality, is problematic. Studies focusing on the existence of a “Glasgow effect” help to demonstrate this.

Part of the challenge, when researching the impact of place effects on health over and above socioeconomic deprivation, is finding comparable populations. This is particularly difficult when a place of interest has extreme levels of deprivation. Existing evidence suggests that Glasgow’s high mortality rates relative to the rest of the Scottish population could largely be explained by its high levels of socioeconomic deprivation. Excess deaths occur in the most deprived areas of Glasgow only, but these are precisely those groups for whom it is most difficult to find comparable populations [15–17]. This problem is well recognised in the literature comparing Glasgow to the rest of Scotland; in a small country there are too few similarly extremely deprived areas which can also be matched on other attributes such as urban and rural classification [17].

Looking internationally for compatible populations has limited potential because of variation in measures of socioeconomic deprivation and because of diverse historical, economic or structural changes that may have impacted population health differently. However, Walsh et al. [8] make comparison between Glasgow and other European regions, with broadly similar post-industrial decline, and conclude that the contextual factors which may aggravate or alleviate the negative consequences of these broad economic trends on population health remain unclear. This reflects the argument that the same contextual processes which influence health may not have the same systematic impact in different places [18].

Comparisons may also be problematic if migration differs between populations and if migration is patterned by deprivation. This is because widening health inequalities may be the result of selective migration processes whereby healthy individuals migrate to healthy areas and away from areas of relatively higher deprivation and poorer health [19, 20]. Although selective migration was not found to account for Glasgow’s increasing mortality when making a within Scotland comparison [19] it may be important for international comparative studies seeking to explain a health difference.

Understanding when mortality in Glasgow changed may provide indicators for the causes of higher mortality. McLoone and Boddy [21] found an increasing difference in mortality between Scotland’s most deprived and least deprived postcode sectors between 1981 and 1991. They attributed the worsening position of Glasgow, relative to Scotland, to the increase in levels of poverty in the city during that time period. This argument was supported by Norman et al. [22] who showed that a rise in premature mortality between 1981 and 2001 was concentrated within the most deprived areas of Scotland, most of which were in Glasgow. Leyland et al. [15] further showed that the increase in deaths in Glasgow was being driven by a rise in premature mortality amongst deprived males. These studies noted that high rates of excess mortality among those living in the most deprived areas was of particular concern because it was in contrast to, and thus partly masked by, improvements at the Scottish national level.

However, the majority of the evidence looking at place effects in Glasgow came from studies using 2001 census data which is no longer the most up-to-date source. Furthermore the notion of a “Glasgow effect” has rarely been explored using life expectancy. Life expectancy is an intuitive measure that allows a clear expression of health differences in number of years, giving it an advantage over the mortality rate. Life expectancy is also a measure of mortality that can be decomposed by age, in order to estimate how much the mortality difference at each age contributes to the total difference in life expectancy at birth [23, 24]. This is important because the risk of dying varies by age, but life expectancy is the aggregate of mortality across all ages. It can therefore mask any differences in mortality experienced by different age groups. Although two populations might share similar life expectancy, the age structure of mortality might differ [23-26]. Understanding age differences in mortality may add to our understanding of inequalities in life expectancy because they highlight more precisely the population groups who are at a disadvantage and to some extent, the processes and aetiological paths leading to mortality differences. If the life expectancy gap between Glasgow and a comparable Scottish population stemmed from specific age groups, it might suggest which processes were responsible for the Glasgow’s poor health record.

As recognised by those studying the “Glasgow effect” there is a danger that the failure to explain health differences between Glasgow and comparable English cities through differences in socioeconomic deprivation levels may lead to a perspective that tackling deprivation is not important for tackling Glasgow’s health problems [27]. The problem has been portrayed in the media, for example, as one of the “Glasgow effect”, rather than of socioeconomic deprivation [28]. In this paper we explore whether differences in life expectancy between Glasgow and a comparator comprising the combined populations of Aberdeen, Dundee and Edinburgh (ADE) are explained by socioeconomic deprivation and whether mortality in Glasgow has a distinct age profile, independent of deprivation which may suggest a distinct “Glasgow effect” amongst Scottish cities.

By calculating and decomposing life expectancy differences the following questions were answered:

1. Is lower life expectancy in Glasgow compared to Aberdeen, Dundee and Edinburgh combined accounted for by Glasgow’s socioeconomic deprivation profile?
2. Does Glasgow have a distinct pattern to the age of death, independent of socioeconomic deprivation in comparison with Aberdeen, Dundee and Edinburgh combined?

## Methods

### Deprivation measures

Deprivation was measured using the income domain of the Scottish Index of Multiple Deprivation 2009 version 2, referred to in the following as SIMD. The SIMD is a small area (datazones) measure of deprivation. There are 6,505 datazones in Scotland with an average population size of 808. About 84 % of the datazones contain between 500 and 999 people [5]. The full SIMD is based on several different domains of social, economic and environmental deprivation but it may not be appropriate to use the full SIMD when evaluating relationships between deprivation and health as the SIMD itself contains measures of mortality and morbidity. Therefore the SIMD income domain only was used. The SIMD income domain is the proportion of people defined as income deprived because they are claiming one or more social security benefits or tax credits triggered by poor economic situation. The income domain is highly correlated with the overall SIMD and contributes 28 % to the overall weight of the full SIMD. The SIMD income domain for the year 2009 was selected because it was based on data from 2009 which was the mid-point for the death and population data being used. Each datazone within the study areas was then attributed to a population weighted decile. Deciles were calculated according to the national distribution of income deprivation, which splits the Scottish population into ten equal groups each containing 10 % of the population. Decile 1 is the 10 % most deprived and decile 10 the 10 % least deprived [29].

### Sensitivity analysis

The sensitivity of the results to the national ranking of deprivation deciles was tested by re-running the analysis using ‘city specific’ deciles. These deciles were established by re-ranking the datazones for Glasgow, Aberdeen, Dundee and Edinburgh populations only. We undertook this comparison because much of the literature on the “Glasgow effect”, especially that comparing Glasgow to cities outside of Scotland, has used such ‘city specific’ deciles [6, 30]. Our preference was to use the deciles created relative to the national range and distribution because this is the more conventional and well-established approach to measuring deprivation [31, 32]. Furthermore the SIMD was created as a single relative measure of national deprivation to be applied to the country as a whole, and was intended to be used to reflect relative deprivation within Scotland [33]. Using deciles calculated from the whole Scottish population to compare Glasgow and ADE means that deprivation is being understood as relative to the notion of ‘Scottish’ deprivation. The substantive conclusions of the research did not depend on which version of the deciles was used – deprivation accounts for the majority of Glasgow’s lower life expectancy. However the ‘city specific’ analysis suggested less of the Glasgow’s lower life expectancy was accounted for by deprivation. In the main paper, we report only results from the national version. All results from the ‘city specific’ analysis are reported in Additional files 1, 2, 3, 4, 5 and 6.

### Population and mortality data

The population of Glasgow was compared to that of Aberdeen, Dundee and Edinburgh (ADE) combined. Each city was defined by its local authority boundary. This comparison population was chosen on the basis that ADE was similar to Glasgow’s population size and represented Scotland’s other major cities.

Deaths and population estimates were aggregated across the years 2007–2011 by sex, age group (0, 1–4, 5–9…85–89, 90+) and SIMD national decile. We smoothed annual fluctuations in the number of deaths, and increased the number of events, by aggregating the data across 5 years. The years 2007–2011 were selected because they represented the most recently available data at the time of the research and population estimates were based on a national census in 2011. The total number of deaths in Aberdeen, Dundee, Edinburgh and Glasgow during this time period was 72,549. Of these deaths, 235 (0.3 %) had to be excluded: 198 deaths could not be matched to a datazone; 35 deaths were matched to a datazone that did not fall within the local authority boundaries used for calculating deprivation deciles with the SIMD; and two infant deaths did not have a sex identified. This left 72,314 deaths to be included in the analysis. Data were obtained and are available on request from National Records of Scotland (http://www.nrscotland.gov.uk/).

### Calculating life expectancy

Sex specific life expectancy at birth with 95 % confidence intervals was calculated using methods implemented in an online Microsoft Excel spreadsheet that is provided by the Max Planck Institute for Demographic Research. Standard life tables are used and 95 % confidence intervals produced using Monte Carlo simulations that are suitable for small populations [34]. Sex specific life expectancy at birth was calculated for (i) each of the two populations (Glasgow and ADE) overall and (ii) for each deprivation decile within each population.

We then quantified the impact of deprivation on life expectancy. First, we calculated a deprivation weighted average life expectancy for Glasgow and ADE by taking an equally weighted average of life expectancies across the deciles within each population. Standard errors for each deprivation decile were pooled to obtain an estimate of 95 % confidence intervals adjusted for deprivation, for both populations separately. Second, we calculated the difference in life expectancy between Glasgow and ADE for overall life expectancy, and then for the deprivation weighted average. Third, we calculated the reduction in life expectancy difference due to deprivation by comparing the difference in life expectancy between Glasgow and ADE from overall life expectancies to the difference from the deprivation weighted average life expectancies.

### Decomposition of life expectancy inequalities

We used Arriaga’s (1984) discrete decomposition method to estimate how much of the total difference in life expectancy at birth between the two populations was contributed by difference in mortality in a specific age group [24, 35]. Number of years contributed to the total difference in life expectancy by each age group has an intuitive interpretation. Decomposition was carried out using StataSE 13.

To determine the influence of deprivation, decomposition was carried out on the difference in life expectancy for the populations overall and then carried out on the differences between the populations across each deprivation decile. The contributions made from each age group for the decompositions carried out across each decile were then averaged by summing the contributions from each age group and dividing by 10. This allowed us to examine if there was an age-specific pattern to mortality in Glasgow that was independent of its deprivation profile.

The research used routinely available data and did not require ethical approval.

## Results

Table 1 shows the comparability of the two populations in terms of socioeconomic deprivation using national deprivation (see Additional file 1 for the same table using ‘city specific’ deprivation). The SIMD ranks all of the 6,505 datazones from most deprived to least deprived, with a rank of 1 being the most deprived and a rank of 6,505 being the least deprived. Table 1 reports the mean income rank of the datazones included in each decile relative to the national distribution. The mean income rank is the sum of all datazones rankings divided by the number of datazones in that decile.

**Table 1:** Comparability of Glasgow and Aberdeen, Dundee and Edinburgh combined when ranking datazones by national income deprivation

	Number of datazones (as % of datazones by location) (SIMD 2009+2)				Mean income rank^a^ (SIMD 2009+2)		Number of income deprived people (as % of population by location) (SIMD 2009+2)				5 year population estimates (as % of population by location) (2007–2011)				5 year number of deaths (as % of population by location by decile) (2007–2011)			
Decile	Glasgow		ADE		Glasgow	ADE	Glasgow		ADE		Glasgow		ADE		Glasgow		ADE	
1(most dep.)	250	36	90	9	276	352	85780	55	28860	25	1,019,818	35	379,813	9	13830	1.4	4042	1.1
2	117	17	79	8	1008	1064	29205	19	17530	15	490,534	17	312,351	8	6821	1.4	4001	1.3
3	67	10	83	8	1701	1688	12515	8	15015	13	268,686	9	330,483	8	3454	1.3	4238	1.3
4	64	9	75	8	2401	2377	10230	7	11975	10	276,051	9	328,472	8	2395	0.9	3536	1.1
5	48	7	75	8	2978	3028	6380	4	9620	8	206,085	7	322,430	8	1719	0.8	3127	1.0
6	46	7	84	8	3649	3688	5160	3	8275	7	210,745	7	345,477	8	1693	0.8	3447	1.0
7	34	5	82	8	4349	4323	2680	2	6610	6	144,076	5	348,543	8	1033	0.7	3244	0.9
8	34	5	96	10	4923	4924	2170	1	5815	5	146,469	5	404,898	10	864	0.6	3903	1.0
9	22	3	124	12	5581	5604	1045	<1	5125	5	103,240	4	500,688	12	726	0.7	3884	0.8
10(least dep.)	12	2	207	21	6092	6242	355	<1	4645	4	43,436	1	864,635	21	276	0.6	6081	0.7
Total	694	100	995	100	1839	3804	155520	100	113470	100	2,909,140	100	4,137,790	100	32811	1.1	39503	1.0

Data obtained and available on request from National Records of Scotland, http://www.nrscotland.gov.uk/

^a^The mean income rank is the average ranking of all datazones included in each decile. E.g. the sum of all datazones rankings divided by the number of datazones in that decile

The two populations had similar distributions of SIMD ranks across each deprivation decile, indicated by the mean rank. Decile 1 and decile 10 were not as closely matched as the other deciles in terms of number of datazones, population size and SIMD rank. The mean SIMD rank for decile 1 for Glasgow is 276 and 352 for ADE; even though there are 160 more datazones within the most deprived decile for Glasgow. This reflects just how extreme Glasgow’s socioeconomic deprivation profile is.

**Fig. 1:**
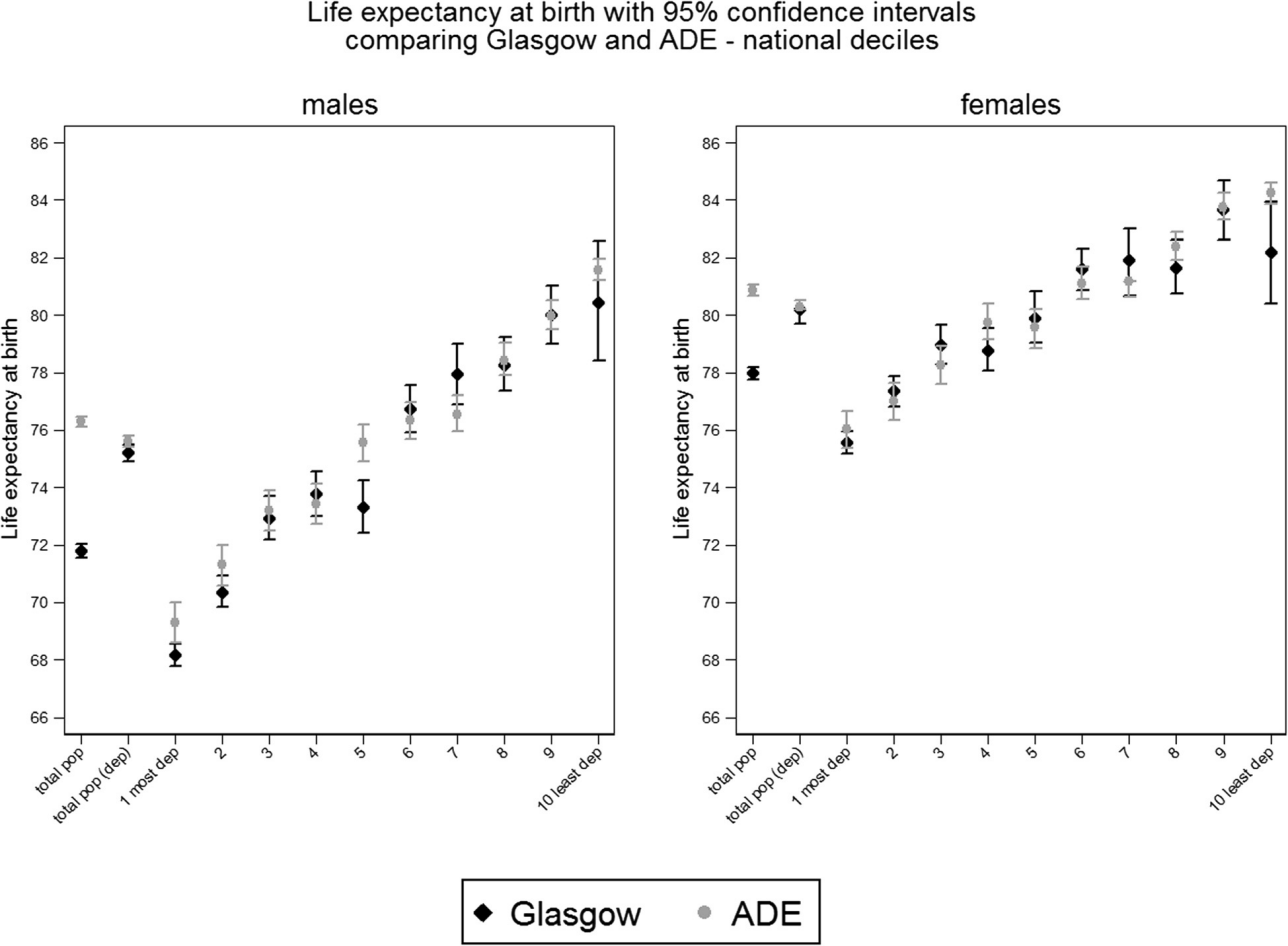
Life expectancy at birth with 95 % confidence intervals by sex, deprivation decile and comparison population. Life expectancy results shown are those calculated for each population weighted income deprivation decile by ranking datazones by national income deprivation

Figure 1 shows total life expectancy of each population before accounting for deprivation (total pop) and after accounting for deprivation (total pop (dep)) at the left end of the X axes. It also shows life expectancy in each income deprivation decile in Glasgow and in ADE. These figures are provided for men and women separately (see Additional file 2 for same figure using ‘city specific’ deprivation). As expected, a deprivation gradient in life expectancy was apparent in both populations. There was a difference of 12.2 years for males and 6.6 years for females between the most deprived and least deprived deciles in Glasgow. In ADE the difference was 12.3 years and 8.2 years for males and females respectively.

Life expectancy, for both males and females, did not differ significantly between Glasgow and ADE in the majority of the deprivation deciles (Fig. 1). The wide confidence intervals for males and females in decile 10 in Glasgow reflect small numbers of least deprived areas and small population size for such affluent people. There was no clear explanation for the slightly lower life expectancy among Glasgow males from decile 5 than among those from decile 4. The difference was not significant and could be due to random fluctuations in the data that remained even after aggregating across 5 years.

Before accounting for deprivation, life expectancy for males in Glasgow was 71.8 (95 % CI 71.6–72.0) and for females it was 78.0 (95 % CI 77.7–78.2). Life expectancy in ADE before accounting for deprivation was 76.3 (95 % CI 76.1–76.5) for males and 80.9 (95 % CI 80.7 – 81.0) for females. This was a difference of 4.5 years for males and 2.9 years for females.

If both groups had the same deprivation profile, life expectancy in Glasgow would be 75.2 (95 % CI 74.9–75.5) for males and 80.2 (95 % CI 79.7–80.5) for females. In ADE life expectancy would be 75.6 (95 % CI 75.4–75.8) for males and 80.3 (95 % CI 80.2–80.5) for females. This was a difference of 0.4 years for males and 0.1 years for females between ADE and Glasgow and meant that 91 % of the difference in life expectancy for males and 93 % of the difference for females was accounted for by deprivation (see Additional file 4 for results using ‘city specific’ deprivation).

**Fig. 2:**
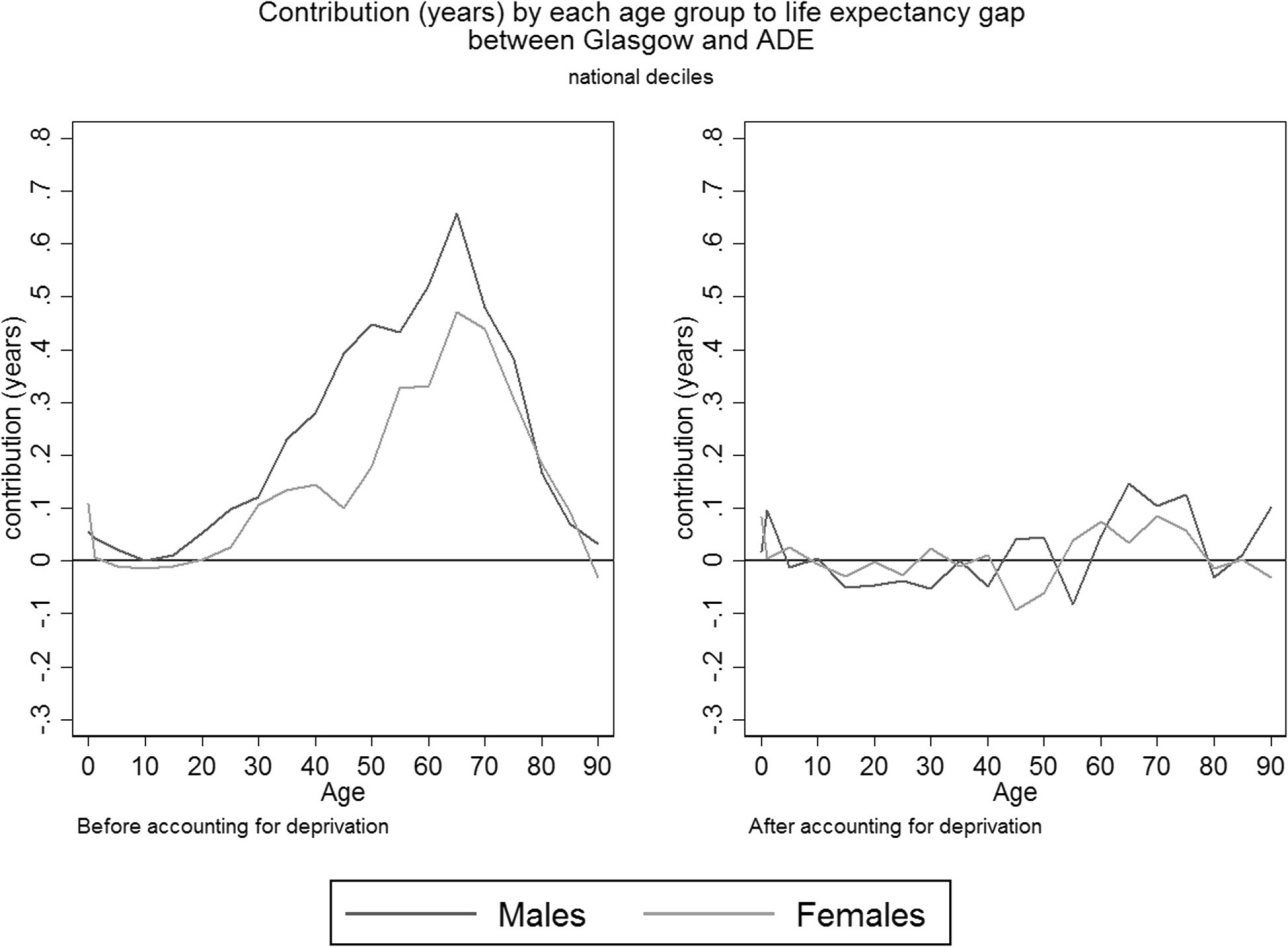
Years contributed by each age group to the difference in life expectancy between Glasgow and ADE before and after accounting for deprivation. After accounting for deprivation is the average contribution (the sum of the contributions from each age group in each decile divided by 10)

Figure 2 shows the decomposition of the life expectancy gap between ADE relative to Glasgow before and after accounting for deprivation (see Additional file 3 for the same figure using ‘city specific’ deprivation). Before accounting for deprivation, a distinct age pattern could be seen with the contribution to the life expectancy gap increasing with age up to age 65 before declining. This pattern is conventional for most populations with a life expectancy of 65 years or more, as death is most common around these ages. In turn, this implies more life years in total can be potentially gained by avoiding the large number of deaths that occur in these ages rather than from the fewer deaths which occur in younger ages and at much older ages. Even though each individual death at an older age loses fewer years of expected life than a death at a younger age, the *total* loss of years from deaths at older ages will be greater because a larger proportion of the population dies at these ages than at younger ages [36]. The pattern showing increasing contributions made up to age 65 was almost completely removed by accounting for deprivation. It suggested that there is no clear age related function of mortality that cuts across the entire Glasgow population once deprivation was accounted for.

## Discussion

The aim of this study was to evaluate whether deprivation could account for lower life expectancy in Glasgow compared to other Scottish cities. Life expectancy was lower for Glasgow compared to the populations of Aberdeen, Dundee and Edinburgh combined (ADE) before accounting for deprivation but the difference was much lower after accounting for deprivation. Deprivation was found to account for over 90 % of the difference in life expectancy between the two populations, although sensitivity analysis using the ‘city specific’ deciles (see Additional files 5 and 6 for comparison) suggested less of this difference was accounted for by deprivation (around 70 %). The unexplained difference which remained may have stemmed from the inability to precisely match levels of deprivation, particularly in the most and least deprived deciles. This would be difficult to achieve because of Glasgow’s extreme deprivation profile within the national context of Scotland.

Previous research suggested that differences between population health in Glasgow and other cities could be accounted for by controlling more accurately for deprivation [17] and that the divergence in mortality between Glasgow and comparable cities may have been driven by an increase in mortality in the most deprived groups only [22, 37]. This study suggests that inequalities in life expectancy between Glasgow and ADE disappear across the majority of deprivation deciles where an equivalent match was achieved. However, it also highlights the difficulty of finding comparable urban populations within Scotland at the most extreme levels of deprivation.

The study has thus provided further evidence for understanding current inequalities in mortality between Scottish cities. When considering Glasgow city’s population, in comparison to other Scottish cities, using national income deprivation deciles the difference which is independent of its extreme deprivation profile (the “Glasgow effect”) is only 10 %. Decomposition showed that there was no distinct pattern of mortality by age that was unique to Glasgow when compared to ADE, after accounting for deprivation. This finding adds to the body of research which argues that deprivation is the most important explanatory factor for Glasgow’s poor health profile within Scotland by demonstrating that there is no age profile of mortality in Glasgow that is independent from socioeconomic deprivation [16, 17, 22].

The study used the most recent, highest quality death and population data, as well as an established measure of socioeconomic deprivation. Prior to this study, most evidence utilised data based on the 2001 census. The income domain of the SIMD is a valid measurement of socioeconomic status and was deemed to be the most appropriate because of its heavy weighting and correlation with the overall SIMD. However, it is possible that the results could change depending on the indicator of socioeconomic deprivation used.

This research used an ecological approach in order to measure deprivation by aggregating datazones into population weighted deprivation deciles according to the Scottish national rankings. Sensitivity analysis was carried out and showed that changing the ranking of datazones to a ‘city specific’ ranking did not alter the substantive conclusions – that deprivation explains the majority of the difference. However, the change in the amount of unexplained difference demonstrates that it can be difficult to find compatible populations at extreme levels of deprivation.

Although an ecological approach for capturing the geographical distribution of deprivation is valid, individual level socioeconomic circumstances may not have been well captured and will have varied within each decile. Whether studying the issue using individual level deprivation would change the result is not clear. Furthermore, there was relatively large uncertainty in life expectancy for certain levels of deprivation (the least deprived in Glasgow for example) because of small population sizes despite pooling data for 5 years. The difficulty in finding comparable populations for Glasgow’s most deprived was emphasised by Dundas et al. [17] who, by using a ‘case control’ method for matching datazones by deprivation, showed a reduction in mortality rate inequalities between Glasgow and the rest of Scotland of up to 57 %. Future research could use this ‘case control’ approach to account for absolute differences in life expectancy.

## Conclusions

This study suggests that over 90 % of excess mortality in Glasgow, relative to comparable Scottish cities, is accounted for by its extreme deprivation profile. In turn, this suggests that area deprivation concentrated within Glasgow may account for a large proportion of Scotland’s mortality disadvantage when making international comparisons. Therefore the main implication of this study is that reducing the high levels of deprivation in Glasgow could reduce inequalities in life expectancy within the city, and between Glasgow and three other major Scottish cities. Tackling deprivation in Glasgow could have positive implications for the health of Scotland as a whole which, in turn, would improve its public health standing in Europe.

## Additional files

Additional file 1:
**Comparability of Glasgow and Aberdeen, Dundee and Edinburgh combined when ranking datazones by ‘city specific’ deprivation.** Table showing the distribution of datazones, mean income rank of decile, number of income deprived, population estimates and deaths for each ‘city specific’ deprivation decile. Datazones in Glasgow, Aberdeen, Dundee and Edinburgh only are re-ranked. (DOCX 26 kb) Additional file 2:
**Life expectancy at birth with 95 % confidence intervals by sex, ‘city specific’ deprivation decile and comparison population.** Plot of life expectancy with 95 % confidence intervals for each ‘city specific’ deprivation decile. (TIFF 1549 kb) Additional file 3:
**Years contributed by each age group to the difference in life expectancy between Glasgow and ADE before and after accounting for ‘city specific’ deprivation.** Age decomposition graph before and after accounting for ‘city specific’ deprivation. (TIFF 1549 kb) Additional file 4:
**Results of ‘city specific’ deprivation analysis.** Life expectancy results using ‘city specific’ deprivation. (DOCX 13 kb) Additional file 5:
**Life expectancy at birth with 95 % confidence intervals comparing national and city specific deciles, males.** Plot of life expectancy with 95 % confidence intervals comparing results using national and ‘city specific’ deprivation deciles, males. Decile 10 in Glasgow was merged with decile 9 when using ‘city specific’ deprivation because of the small population size. (TIFF 1549 kb) Additional file 6:
**Life expectancy at birth with 95 % confidence intervals comparing national and city specific deciles, females.** Plot of life expectancy with 95 % confidence intervals comparing results using national and ‘city specific’ deprivation deciles, females. Decile 10 in Glasgow was merged with decile 9 when using ‘city specific’ deprivation because of the small population size. (TIFF 1549 kb)
