# Supplementary material for: How much of the difference in life expectancy between Scottish cities does deprivation explain?
Source: BMC Public Health. 2015 Oct 16;15:1057. doi: 10.1186/s12889-015-2358-1 (PMC4608116; doi:10.1186/s12889-015-2358-1)
Supplement: Additional file 1: — Comparability of Glasgow and Aberdeen, Dundee and Edinburgh combined when ranking datazones by ‘city specific’ deprivation. Table showing the distribution of datazones, mean income rank of decile, number of income deprived, population estimates and deaths for each ‘city specific’ deprivation decile. Datazones in Glasgow, Aberdeen, Dundee and Edinburgh only are re-ranked. (DOCX 26 kb) [file 12889_2015_2358_MOESM1_ESM.docx]

| Comparability of Glasgow and Aberdeen, Dundee and Edinburgh combined when ranking datazones by ‘city specific’ income deprivation | | | | | | | | | | | | | | | | | | |
| --- | --- | --- | --- | --- | --- | --- | --- | --- | --- | --- | --- | --- | --- | --- | --- | --- | --- | --- |
|  | Number of datazones  (as % of datazones by location)  (SIMD 2009+2) | | | | Mean income rank*  (SIMD 2009+2) | | Number of income deprived people  (as % of population by location)  (SIMD 2009+2) | | | | 5 year Population estimates  (as % of population by location)  (2007-2011 census derived) | | | | 5 year number of deaths  (as % of population by location and by decile)  (2007-2011) | | | |
| Decile | Glasgow | | ADE | | Glasgow | ADE | Glasgow | | ADE | | Glasgow | | ADE | | Glasgow | | ADE | |
| 1(most dep) | 140 | 20 | 40 | 4 | 124 | 163 | 52,290 | 34 | 14,650 | 13 | 565,218 | 19 | 173,528 | 4 | 7375 | 1.3 | 1788 | 1.0 |
| 2 | 121 | 17 | 51 | 5 | 490 | 506. | 36,615 | 24 | 14,410 | 13 | 501,980 | 17 | 209,476 | 5 | 7181 | 1.4 | 2269 | 1.1 |
| 3 | 101 | 15 | 71 | 7 | 1,026 | 1,042 | 25,110 | 16 | 16,050 | 14 | 425,969 | 15 | 284,893 | 7 | 5903 | 1.4 | 3631 | 1.3 |
| 4 | 77 | 11 | 100 | 10 | 1,702 | 1,703 | 14,395 | 9 | 18,010 | 16 | 307,072 | 11 | 397,180 | 10 | 3829 | 1.2 | 5086 | 1.3 |
| 5 | 80 | 12 | 82 | 8 | 2,523 | 2,499 | 12,165 | 8 | 12,785 | 11 | 340,897 | 12 | 367,827 | 9 | 3131 | 0.9 | 3909 | 1.1 |
| 6 | 57 | 8 | 105 | 11 | 3,341 | 3,299 | 7,160 | 5 | 12,115 | 11 | 261,274 | 9 | 441,023 | 11 | 1830 | 0.7 | 4361 | 1.0 |
| 7 | 49 | 7 | 118 | 12 | 4,184 | 4,177 | 4,160 | 3 | 9,795 | 9 | 210,045 | 7 | 489,682 | 12 | 1683 | 0.8 | 4573 | 0.9 |
| 8 | 41 | 6 | 122 | 12 | 4,972 | 5,009 | 2,525 | 2 | 7,145 | 6 | 174,329 | 6 | 513,218 | 12 | 1069 | 0.6 | 4935 | 1.0 |
| 9 | 25 | 4 | 141 | 14 | 5,787 | 5,757 | 1,085 | <1 | 5,245 | 5 | 121,824 | 4 | 575,371 | 14 | 800 | 0.7 | 4165 | 0.7 |
| 10(least dep.)** | 2 | <1 | 165 | 17 | 6,418 | 6,311 | 15 | <1 | 3,265 | 3 | 532 | <1 | 685,592 | 17 | 10 | 1.9 | 4786 | 0.7 |
| Total | 694 | 100 | 995 | 100 | 1,839 | 3,804 | 155,520 | 100 | 113,470 | 100 | 2,909,140 | 100 | 4,137,790 | 100 | 32811 | 1.1 | 39503 | 1.0 |
| Data obtained and available on request from National Records of Scotland, <http://www.nrscotland.gov.uk/>  *The mean income rank is the average ranking of all datazones included in each decile. E.g. the sum of all datazones rankings divided by the number of datazones in that decile. | | | | | | | | | | | | | | | | | | |
