# Supplementary figures and images for: How much of the difference in life expectancy between Scottish cities does deprivation explain?
Source: BMC Public Health. 2015 Oct 16;15:1057. doi: 10.1186/s12889-015-2358-1 (PMC4608116; doi:10.1186/s12889-015-2358-1)

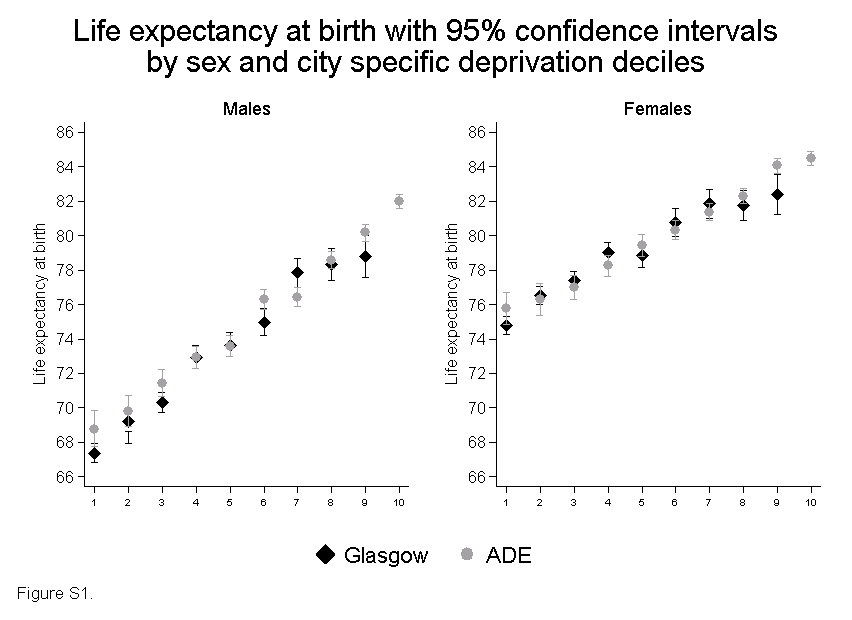

Supplement: Additional file 2: — Life expectancy at birth with 95 % confidence intervals by sex, ‘city specific’ deprivation decile and comparison population. Plot of life expectancy with 95 % confidence intervals for each ‘city specific’ deprivation decile. (TIFF 1549 kb) [file 12889_2015_2358_MOESM2_ESM.tiff]

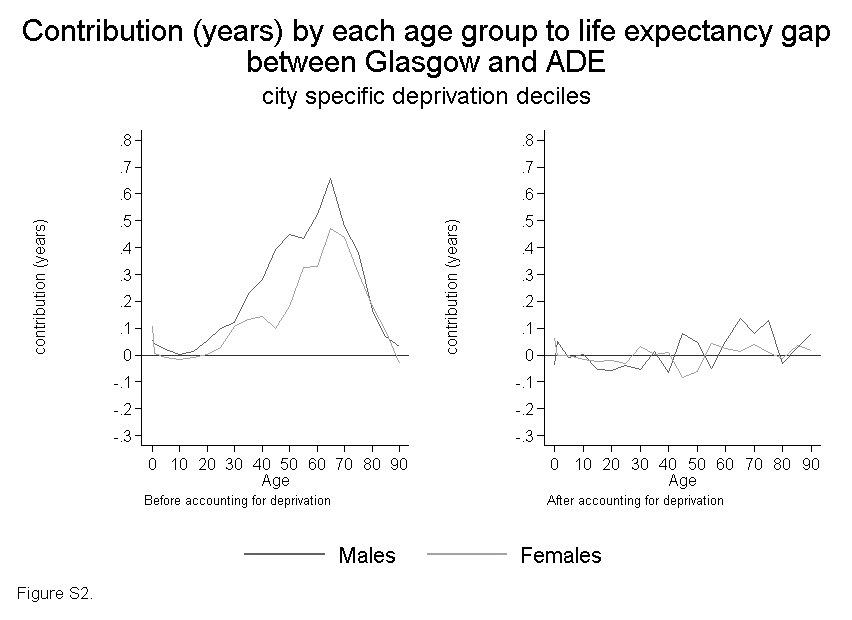

Supplement: Additional file 3: — Years contributed by each age group to the difference in life expectancy between Glasgow and ADE before and after accounting for ‘city specific’ deprivation. Age decomposition graph before and after accounting for ‘city specific’ deprivation. (TIFF 1549 kb) [file 12889_2015_2358_MOESM3_ESM.tiff]

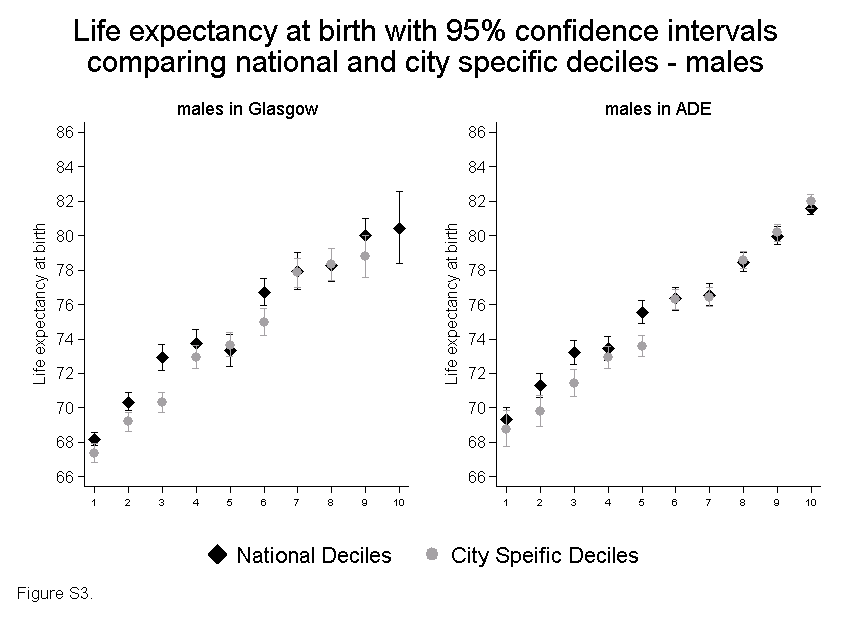

Supplement: Additional file 5: — Life expectancy at birth with 95 % confidence intervals comparing national and city specific deciles, males. Plot of life expectancy with 95 % confidence intervals comparing results using national and ‘city specific’ deprivation deciles, males. Decile 10 in Glasgow was merged with decile 9 when using ‘city specific’ deprivation because of the small population size. (TIFF 1549 kb) [file 12889_2015_2358_MOESM5_ESM.tiff]

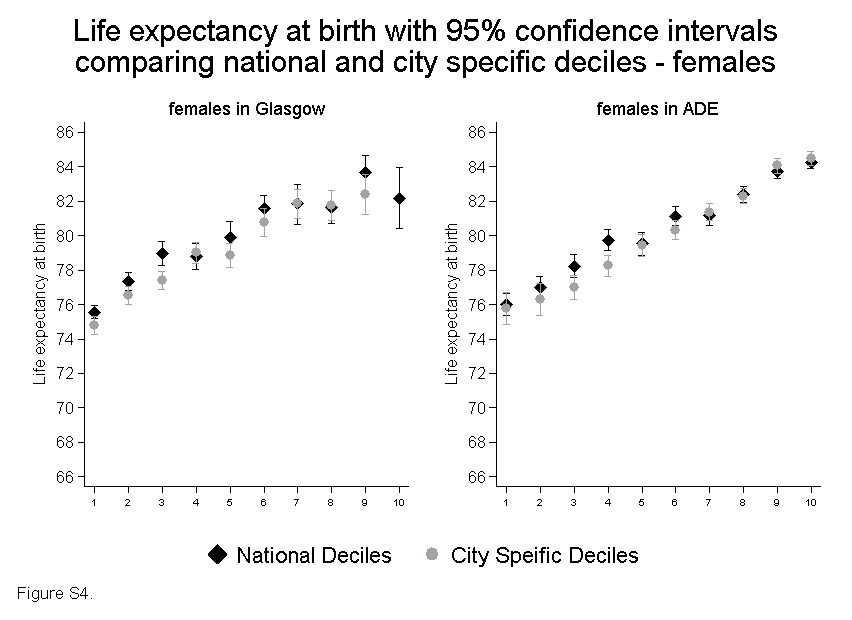

Supplement: Additional file 6: — Life expectancy at birth with 95 % confidence intervals comparing national and city specific deciles, females. Plot of life expectancy with 95 % confidence intervals comparing results using national and ‘city specific’ deprivation deciles, females. Decile 10 in Glasgow was merged with decile 9 when using ‘city specific’ deprivation because of the small population size. (TIFF 1549 kb) [file 12889_2015_2358_MOESM6_ESM.tiff]
