# Supplementary material for: How much of the difference in life expectancy between Scottish cities does deprivation explain?
Source: BMC Public Health. 2015 Oct 16;15:1057. doi: 10.1186/s12889-015-2358-1 (PMC4608116; doi:10.1186/s12889-015-2358-1)
Supplement: Additional file 4: — Results of ‘city specific’ deprivation analysis. Life expectancy results using ‘city specific’ deprivation. (DOCX 13 kb) [file 12889_2015_2358_MOESM4_ESM.docx]

**‘City Specific’ Deprivation Deciles**

Before accounting for deprivation, life expectancy for males in Glasgow was 71.8 (95% CI 71.6-72.0) and for females it was 78.0 (95% CI 77.7-78.2). Life expectancy in ADE before accounting for deprivation was 76.3 (95% CI 76.1-76.5) for males and 80.9 (95% CI 80.7 – 81.0) for females. This was a difference of 4.5 years for males and 2.9 years for females.

Once deprivation was accounted for, life expectancy in Glasgow was 73.7 (95% CI 73.5–74.0) for males and 79.3 (95% CI 79.0-79.5) for females. In ADE life expectancy was 75.0 (95% CI 74.8-75.2) for males and 79.9 (95% CI 79.7-80.1) for females. This was a difference of 1.3 years for males and 0.6 years for females between ADE and Glasgow and means that 71% of the difference in life expectancy for males and 75% of the difference for females is accounted for by deprivation.
